# Genomic DNA Methylation in Diabetic Chronic Complications in Patients With Type 2 Diabetes Mellitus

**DOI:** 10.3389/fendo.2022.896511

**Published:** 2022-06-29

**Authors:** Xixi Wang, Wenhong Yang, Yunyan Zhu, Shiyu Zhang, Miao Jiang, Ji Hu, Hong-Hong Zhang

**Affiliations:** ^1^ Department of Endocrinology, The Second Affiliated Hospital, Soochow University, Suzhou, China; ^2^ Department of Nursing, The Second Affiliated Hospital, Soochow University, Suzhou, China

**Keywords:** type 2 diabetes, genomic DNA methylation, chronic complications, diabetic peripheral neuropathy, LC-MS/MS

## Abstract

**Aim:**

To explore the relationship between genomic DNA methylation and diabetic chronic complications.

**Methods:**

299 patients with type 2 diabetes mellitus (T2DM) hospitalized in the Second Affiliated Hospital of Soochow University were enrolled. We divided the patients into different complications groups and corresponding non-complication groups. Clinical and biochemical parameters were compared between the two groups. The level of genomic DNA methylation in leukocytes was determined by high-performance liquid chromatography-tandem mass spectrometry.

**Results:**

(1) Age, duration of diabetes, creatinine (Cr), blood urea nitrogen (BUN), genomic DNA methylation, 24- hour urine total protein (24-hUTP), and intima-media thickness (IMT) were significantly higher in the carotid plaque (CP) group. Waist-to-hip ratio (WHR), body mass index (BMI), estimated glomerular- filtration rate (eGFR), and albumin (Alb) were significantly lower in the CP group. Gender, age and BMI were the influencing factors of CP. (2) Age, duration, Cr, BUN, urinary microalbumin creatinine ratio (UACR), systolic blood pressure (SBP), TCSS, and 24- hUTP were significantly higher in the diabetic retinopathy (DR) group. eGFR, 2h postprandial C- peptide, and Alb were lower in the DR group. Age, duration, Cr, Alb, SBP, and the presence of DN were the influencing factors of DR. (3) Age, duration, HbA1c, BUN, TCSS, SBP, and IMT(R) were significantly higher in the diabetic nephropathy (DN) group. 2h postprandial C-peptide, and Alb were lower in the DN group. HbA1c, BUN, DR, and HBP were the influencing factors of DN. (4) Age, duration, total cholesterol (TC), low-density lipoprotein (LDL-C), triglyceride (TG), Cr, BUN, uric acid (UA), and SBP were significantly higher in the diabetic peripheral neuropathy (DPN) group. The level of genomic DNA methylation and eGFR were significantly lower in the DPN group. Age, duration, LDL-C, UA, the presence of DR, and the genomic DNA methylation level were the influencing factors for DPN. Incorporating the level of genomic DNA methylation into the prediction model could improve the ability to predict DPN on the basis of conventional risk factors.

**Conclusion:**

Low level of genomic DNA methylation is a relatively specific risk factor for DPN in patients with T2DM and not a contributing factor to the other chronic complications.

## 1 Introduction

The prevalence of type 2 diabetes mellitus (T2DM) is supposed to increase year by year, with the number of people suffering from diabetes likely to reach 147.2 million by 2045 (1). The symptoms of diabetes are usually not so obvious at the early stage that many patients are diagnosed when the complications have occurred. Chronic hyperglycemia leads to serious life-threatening diseases, including retinopathy, nephropathy, neuropathy and atherosclerosis (AS). Up till now, there is no effective treatment for these diabetes complications. Early intervention of the risk factors for diabetic complications is thought to be an effective strategy to delay and prevent the complications of diabetes.

Epigenetic modifications include DNA methylation, regulatory RNAs, and posttranslational histone tail modifications, such as methylation, phosphorylation, acetylation. DNA methylation refers to transferring a methyl from S-adenosylmethionine (SAM) to cytosine on a CpG dinucleotide by DNA methyltransferase (DNMTs). DNA methylation is associated with the abnormal gene expression, DNA repair, genomic instability, and changes in genetic traits. It alters the structure of chromatin and gene expression. It is reported that the role of DNA methylation is very important in T2DM and its chronic complications (2).

## 2 Research Design and Methods

### 2.1 Research Design

According to admission criteria, we collected and analyzed the clinical data, laboratory tests, and blood samples from T2DM patients. The patients were divided into diabetic nephropathy (DN) group and non-DN group, diabetic retinopathy (DR) group and non-DR group, diabetic peripheral neuropathy (DPN) group and non-DPN group, and carotid plaque (CP) group and non-carotid plaque (non-CP) group, according to the results of urinary albumin creatinine ratio (UACR), fundus photographs, Toronto Clinical Scoring System (TCSS) scores, and ultrasound scan of carotid artery, respectively.

### 2.2 Study Protocol

A total of 299 T2DM patients hospitalized in the endocrine department of the Second Affiliated Hospital of Soochow University were enrolled in this study. T2DM was diagnosed using WHO diagnostic criteria published in 1999. The following diseases or conditions were excluded: symptomatic cardiovascular disease, previous surgery to the carotid arteries, severe acute and chronic inflammation, chronic alcohol addiction, cancer, and other severe disorders. In addition, patients suffering from hyperosmotic, hyperthyroidism, acute complications of diabetes, including hyperglycemic hyperosmolar syndrome (HHS), diabetic ketoacidosis (DKA), and lactic acidosis, were also excluded. During interviews with the subjects, physicians or the coordinators completed case report forms, recording the disease profile of diabetes, and related treatment histories including the medicines they took. The protocol for this study was authorized by the Institutional Review Board of the Second Affiliated Hospital of Soochow University, and the authorized registration number is (2016) Ethics Review No.K11. Each patient was given complete information about this trial and was required to sign a written consent form. The sample data was saved electronically in a dedicated computer with a password only accessible by researchers, ensuring that the patients’ personal information was kept private. The samples were kept in a special biological sample storage refrigerator, and the key of refrigerator was held by the principal investigators. The blood samples could only be measured in the present study. The medical records of all the patients were retained in the hospital.

### 2.3 Clinical and Biochemical Measurements

Anthropometric measurements including body weight, height, measured with light clothes and bare feet, and blood pressure (BP), were performed on each patient. Hypertension refers to the measurement of blood pressure ≥140/90 mmHg in resting state. If the patient has previously been diagnosed with hypertension and is currently receiving antihypertensive medication, he or she will still be diagnosed with hypertension even if the measured blood pressure is less than 140/90mmHg. The formula: BMI (kg/m²) = weight(kg)/height(m)², was used to calculate body mass index (BMI). The waist-to-hip ratio (WHR) was obtained by dividing waist circumference (cm) by hip circumference (cm).

The “gold standard” for diagnosing DPN is the electroneuromyography testing. However, it is not suitable for large-scale screening of diabetic patients in practice. TCSS is well applied in early screening and diagnosis of DPN, because when the TCSS scores ≥ 6, the sensitivity and specificity are more than 70% (3). In the present study, TCSS was used to evaluate DPN, and the method was as previously described in details (4, 5). A technician with over 10 years of experience in neurological examinations handled the query and testing. Ask each patient for pain sensation (eg, stabbing, burning, or shock-like pain), numbness, tingling and weakness in the feet, similar symptoms on the upper limbs, and instability when walking. Sensory testing was performed on the first toe and the results were recorded as normal or abnormal. Ask patients how their toes feel when stimulated by needles, light touch, instruments of different temperatures, tuning forks, and how their joints positions feel. Lower limbs nerve reflexes, including knee reflex and ankle reflex, were tested separately. TCSS score was a continuous variable ranging from a minimum of 0 (no neuropathy) to a maximum of 19 points. 6 points were obtained from symptoms, 5 from sensory testing of distal toes, and 8 from lower limb reflexes. Patients with TCSS scores ≥ 6 points were assigned to the DPN group, and the scores<6 points were regarded as no DPN.

The blood samples were collected after all patients fasted for at least 8 hours. The fully automated blood cell analyzer (Sysmex kx-21N, Japan) was used to determine blood routines. Biochemical parameters, such as total cholesterol (TC), low-density lipoprotein (LDL-C), high-density lipoprotein (HDL-C), triglyceride (TG), creatinine (Cr), uric acid (UA), blood urea nitrogen (BUN), serum albumin (Alb), C-reactive protein (CRP), 24h urine total protein (24-hUTP) and urinary microalbumin creatinine ratio(UACR) were tested using an automated biochemical instrument (Cobas800-c702, Roche, Basel, Switzerland). Roche Cobas e601 (Roche Diagnostic, Germany) was used for fasting C-peptides and 2 hours postprandial C-peptides. The Bole D100 glycation hemoglobin meter (Bole, USA) was used for glycosylated hemoglobin. eGFR was calculated with the modified MDRD equation. Intima-media thickness (IMT) of the carotid artery and carotid plaque were tested by the Korea GE Color Ultrasound Diagnostic Instrument (LOGIQ S7). Diagnosis of atherosclerosis was according to the 2009 Chinese Physicians Association Ultrasound Division Vascular Ultrasound Guidelines. The normal IMT of the carotid artery is less than 1.0mm. The diagnostic criteria for thickening of the carotid artery are 1.0mm≤ IMT ≤ 1.5mm. The diagnostic criteria for atherosclerotic plaque formation are that the carotid intimal thickening exceeds 50% of the peripheral IMT or protrudes into the vascular lumen, and IMT ≥ 1.5mm (6).

### 2.4 Genomic DNA Methylation Detection

The HiPure Blood DNA Kits (D3111, Magen) were used to extract DNA from white blood cells. As previously described in details (4), LC-MS/MS (Agilent 1260-API 4000, USA) was used to determine the level of genomic DNA methylation. DNA was cracked with 200μl of 99% formic acid at 140°C for 90 minutes, and fragment was suspended with 200μl water for next analysis. The volume of injection was 10μl. Mass spectrometric measurement was carried out on an API 4000 instrument (SCIEX, Ontario, Canada). We ran the tandem mass spectrometers with multiple reaction monitoring modes, m/z 112.1→95.1 and m/z 126.1→109.1 for Cyt and 5-mCyt, respectively. 5-methylcytosine hydrochloride (5-mCyt) and Cytosine (Cyt) were bought from Sigma-Aldrich (MKBX8310V and MKBQ8997V, USA). The genomic DNA methylation level was calculated as the percentage of DNA methylation using the following formula: DNA methylation%= 5-mCyt/(5-mCyt + Cyt)× 100%.

### 2.5 Statistical Methods

Patients’ characteristics were compared by presence of DPN or not, presence of DR or not, presence of DN or not and presence of CP or not. All data were shown as means ± standard error (SEM). We used Software SPSS 24.0 for data analysis. Normality was checked for all data prior to analysis. Two-sample *t*-test, Mann-Whitney *U* test, or *χ*2 test were used to compare the differences between the two groups. Spearman correlation analysis was used to analyze the associations among clinical and biochemical factors, the level of genomic DNA methylation, and TCSS scores. Multiple logistic regressions analysis was used to evaluate the influencing factors of different diabetic chronic complications. Multiple linear regression analysis was used to evaluate the influencing factors of genomic DNA methylation. A *p* value less than 0.05 was considered statistically significant.

## 3 Results

### 3.1 Patients

A total of 299 patients with T2DM were recruited in the present study. Of the 299 diabetic patients, average age was 54.43 years with 33.78% being female, and average BMI was 25.56 kg/m^2^.

### 3.2 Diabetic Complications

#### 3.2.1 Carotid Plaque

As shown in **Table 1**, the patients were divided into the CP group and the non-CP group. Age, duration of diabetes, Cr, BUN, 24-hUTP, IMT and the level of genomic DNA methylation were much higher in the CP group (**p < 0.01, *p < 0.05, compared with non-CP, two-sample t-test and Mann-Whitney *U* test). BMI, WHR, Alb and eGFR were significantly lower in the CP group (**p < 0.01, *p < 0.05, compared with non-DN, Mann-Whitney *U* test). Male patients and smokers were more likely to develop CP (**p < 0.01, *p < 0.05, *χ*2 test). In the multiple logistic regression analysis, we set the presence or absence of CP as the dependent variable and the other fourteen variables as independent variables. The findings revealed that gender, age and BMI were still independently associated with the risk of CP, while duration, WHR, Cr, BUN, eGFR, the level of genomic DNA methylation, Alb, 24-hUTP and smoking were no longer related (**Table 2**, **p < 0.01, *p < 0.05, *p*>0.05).

**Table 1:** Clinical and biochemical characteristics in CP group and non-CP group.

Variables	Non-CP (n=143)	CP (n=156)	*p* Value
Gender (M: F)	86: 57	112: 44	0.030^*^
Age (yr)	49.700 ± 1.056	58.780 ± 1.006	0.000^**^
Duration (yr)	6.704 ± 0.501	9.211 ± 0.578	0.013^*^
BMI (kg/m^2^)	26.592 ± 0.375	24.867 ± 0.293	0.000^**^
WHR	0.958 ± 0.006	0.942 ± 0.005	0.029^*^
FPG (mmol/L)	9.095 ± 0.381	8.983 ± 0.355	0.775
Fasting C-peptide(ng/ml)	1.909 ± 0.098	1.827 ± 0.082	0.822
C-peptide 2h postprandial(ng/ml)	4.585 ± 0.274	4.369 ± 0.265	0.595
HbA1c (%)	8.864 ± 0.186	8.954 ± 0.209	0.924
TG (mmol/L)	2.017 ± 0.151	1.871 ± 0.129	0.713
TC (mmol/L)	4.786 ± 0.089	4.752 ± 0.098	0.799
LDL-C (mmol/L)	2.814 ± 0.074	2.887 ± 0.076	0.493
HDL-C (mmol/L)	1.073 ± 0.026	1.066 ± 0.025	0.648
Cr (umol/L)	65.969 ± 1.911	72.213 ± 2.286	0.030^*^
UA (umol/L)	337.270 ± 9.072	322.010 ± 6.911	0.177
BUN (mmol/L)	5.518 ± 0.177	5.953 ± 0.162	0.030^*^
eGFR (ml/min/1.73m^2^)	118.615 ± 3.538	105.314 ± 2.846	0.003^**^
UACR (mg/g)	210.860 ± 74.493	179.936 ± 35.951	0.448
CRP (mg/L)	8.158 ± 1.243	5.853 ± 0.192	0.749
Genomic DNA methylation (%)	9.638 ± 0.731	10.885 ± 0.624	0.042^*^
TCSS	6.350 ± 0.405	7.430 ± 0.376	0.074
Alb(g/L)	42.535 ± 0.401	41.292 ± 0.356	0.011^*^
24h-UTP (g/24h urine)	118.243 ± 48.737	163.487 ± 41.635	0.002^**^
IMT(L)(mm)	0.686 ± 0.015	0.851 ± 0.002	0.000^**^
IMT(R)(mm)	0.671 ± 0.012	0.789 ± 0.013	0.000^**^
SBP (mmHg)	137.221 ± 1.337	142.528 ± 1.906	0.084
DBP (mmHg)	82.228 ± 0.742	79.924 ± 0.926	0.055
DR (Y: N)	24: 118	35: 121	0.231
DPN (Y: N)	31: 37	66: 48	0.107
DN (Y: N)	24: 119	37: 119	0.137
Smoke (Y: N)	34: 109	54: 102	0.040^*^
HBP (Y: N)	68: 75	87: 69	0.155

CP, carotid plaque; Duration, duration of diabetes; BMI, body mass index; WHR, waist-to-hip ratio; FPG, fasting plasma glucose; HbA1c, glycated hemoglobin A1c; TG, triglycerides; TC, total cholesterol; LDL-c, low density lipoprotein cholesterol; HDL-c, high density lipoprotein cholesterol; Cr, creatinine; UA, uric acid; BUN, blood urea nitrogen; eGFR, estimated glomerular filtration rate; UACR, urinary albumin creatinine ratio; CRP, c-reactive protein; TCSS, Toronto Clinical Scoring System; Alb, albumin; 24h-UTP, 24-hour urine total protein; IMT(L), intima-media thickness(left); IMT(R), intima-media thickness(right); SBP, systolic blood pressure; DBP, diastolic blood pressure; DR, diabetic retinopathy; DPN, diabetic peripheral neuropathy; DN, diabetic nephropathy; HBP, high blood pressure. Data are means ± SEM or numbers of patients. The level of DNA methylation, duration of diabetes, BMI, WHR, fasting C-peptide, C-peptide 2h postprandial, HbA1c, FPG, CRP, TG, Cr, BUN, Alb, eGFR, 24h-UTP, UACR and IMT were non-parametric and Mann-Whitney U test was used to compare them between two groups. **p<0.01, *p < 0.05. p values for differences between the both groups were obtained by two-sample t-test, Mann-Whitney U test or χ^2^ test.

**Table 2:** CP as dependent variable in multiple logistic regression analysis.

	B	Std.Error	*p* Value	OR	95% Confidence Interval for OR	
					Lower Bound	Upper Bound
Constant	2.146	1.921	0.264	8.553	–	–
Gender	-1.454	0.417	0.000**	0.234	0.103	0.529
Age (yr)	0.083	0.018	0.000**	1.086	1.048	1.126
BMI (kg/m^2^)	-0.173	0.062	0.000**	0.841	0.745	0.949

In multiple logistic regression analysis, carotid plaque, as dependent variable, and the other 14 variables, gender, duration, age, BMI, WHR, Cr, BUN, eGFR, the level of DNA methylation, Alb, 24-hUTP, smoking, IMT(L) and IMT(R), as independent variables, were included in the same model. Only 3 variables, gender, age and BMI were the risk factors of CP (**p < 0.01).

#### 3.2.2 Diabetic Retinopathy

As shown in **Table 3**, the patients were divided into the DR group and the non-DR group. Age, duration, Cr, BUN, UACR, SBP, TCSS and 24-hUTP was much higher in the DR group (**p < 0.01, *p < 0.05, compared with non-DR, two-sample t-test and Mann-Whitney *U* test). Alb, 2h postprandial C-peptide and eGFR was significantly lower in the DR group (**p < 0.01, *p < 0.05, compared with non-DR, Mann-Whitney *U* test). The presence of DR was associated with the presence of DN and DPN (**p < 0.01, *p < 0.05, *χ*2 test). In the multiple logistic regression analysis, we set the presence or absence of DR as the dependent variable and the other thirteen variables as the independent variables. The findings revealed that age, duration, Alb, Cr, SBP and the presence of DN were still independently associated with the risk of DR, while 2h postprandial C-peptide, BUN, eGFR, UACR, TCSS, 24-hUTP and DPN were no longer related (**Table 4**, **p < 0.01, *p < 0.05, *p*>0.05).

**Table 3:** Clinical and biochemical characteristics in DR group and non-DR group.

Variables	Non-DR (n=239)	DR (n=59)	*p* Value
Gender (M: F)	160: 79	37: 22	0.538
Age (yr)	53.640 ± 0.887	57.790 ± 1.506	0.013^*^
Duration (yr)	7.049 ± 0.412	12.147 ± 0.901	0.000^**^
BMI (kg/m^2^)	25.712 ± 0.278	25.193 ± 0.466	0.365
WHR	0.951 ± 0.005	0.941 ± 0.007	0.467
FPG (mmol/L)	8.997 ± 0.278	9.302 ± 0.691	0.660
Fasting C-peptide (ng/ml)	1.924 ± 0.072	1.674 ± 0.131	0.080
C-peptide 2h postprandial (ng/ml)	4.683 ± 0.219	3.723 ± 0.355	0.025^*^
HbA1c (%)	8.910 ± 0.159	8.849 ± 0.305	0.793
TG (mmol/L)	1.942 ± 0.110	1.955 ± 0.231	0.613
TC (mmol/L)	4.733 ± 0.745	4.922 ± 0.153	0.263
LDL-C (mmol/L)	2.829 ± 0.059	2.955 ± 0.123	0.348
HDL-C (mmol/L)	1.051 ± 0.019	1.134 ± 0.047	0.095
Cr (umol/L)	66.064 ± 1.319	82.047 ± 5.175	0.022^*^
UA (umol/L)	325.70 ± 6.412	343.930 ± 12.084	0.200
BUN (mmol/L)	5.529 ± 0.117	6.655 ± 0.363	0.007^**^
eGFR (ml/min/1.73m^2^)	115.311 ± 2.431	96.739 ± 5.578	0.001^**^
UACR (mg/g)	129.135 ± 42.454	466.765 ± 109.892	0.000^**^
CRP (mg/L)	7.283 ± 0.742	5.544 ± 0.747	0.852
Genomic DNA methylation	10.290 ± 0.539	10.600 ± 1.091	0.781
TCSS	6.420 ± 0.303	9.020 ± 0.611	0.000^**^
Alb (g/L)	42.464 ± 0.280	39.843 ± 0.6401	0.000^**^
24h-UTP (g/24h urine)	86.212 ± 28.764	343.518 ± 106.389	0.002*
IMT (L) (mm)	0.767 ± 0.164	0.781 ± 0.291	0.544
IMT (R) (mm)	0.721 ± 0.010	0.768 ± 0.250	0.071
SBP (mmHg)	137.502 ± 1.231	149.161 ± 2.993	0.000^**^
DBP (mmHg)	80.538 ± 0.632	82.714 ± 1.591	0.208
DN (Y: N)	67: 172	36: 23	0.000^**^
DPN (Y: N)	92: 73	11: 36	0.000^**^
CP (Y: N)	121: 118	35: 24	0.231
HBP (Y: N)	118: 121	36: 23	0.109

Data are means ± SEM or numbers of patients. The level of DNA methylation, duration of diabetes, WHR, fasting C-peptide, C-peptide 2h postprandial, HbA1c, FPG, CRP, TG, Cr, BUN, Alb, eGFR, 24h-UTP, UACR and IMT were non-parametric and we used Mann-Whitney U test to compare them between two groups. ^*^p < 0.05, ^**^p < 0.01. p values for differences between the both groups were obtained by two-sample t-test, Mann-Whitney U test or χ^2^ test.

**Table 4:** DR as dependent variable in multiple logistic regression analysis.

	B	Std.Error	*p* Value	OR	95% Confidence Interval for OR	
					Lower Bound	Upper Bound
Constant	1.033	3.004	0.731	2.809	–	–
Age (yr)	0.024	0.025	0.017**	0.941	0.895	0.989
Cr (umol/L)	0.019	0.007	0.010^*^	1.019	1.004	1.034
Alb (g/L)	-0.171	0.060	0.005**	0.843	0.749	0.949
SBP (mmHg)	0.038	0.013	0.004**	1.039	1.012	1.066
DN	0.967	0.484	0.046*	2.629	1.018	6.794

In multiple logistic regression analysis, DR, as dependent variable, and the other 13 variables, Age, duration, 2h postprandial C-peptide, Cr, BUN, eGFR, UACR, TCSS, Alb, 24-hUTP, SBP, the presence of DN and the presence of DPN, as independent variables, were included in the same model. 6 variables, age, duration, Cr, Alb, SBP and DN were the risk factors of DR (**p < 0.01, *p < 0.05).

#### 3.2.3 Diabetic Nephropathy

As shown in **Table 5**, the patients were divided into the DN group and the non-DN group. Age, duration, HbA1c, BUN, TCSS, SBP and IMT(R) were significantly higher in the DN group (**p < 0.01, *p < 0.05, compared with non-DN, two-sample t-test and Mann-Whitney *U* test). 2h postprandial C-peptide and Alb were much lower in DN group (**p < 0.01, *p < 0.05, compared with non-DN, Mann-Whitney *U* test). DN was associated with the presence of DR, DPN and HBP (**p < 0.01, *p < 0.05, *χ*2 test). In the multiple logistic regression analysis, we set the presence or absence of DN as a dependent variable and the other twelve variables as independent variables. The findings revealed that HbA1c, BUN, the presence of DR and HBP were still independently associated with the risk of DN, while age, duration, TCSS, Alb, SBP, IMT(R), 2h postprandial C-peptide and the presence of DPN were no longer related (**Table 6**, **p < 0.01, *p < 0.05, *p*>0.05).

**Table 5:** Clinical and biochemical characteristics in DN group and non-DN group.

Variables	Non-DN (n=195)	DN (n=104)	*p* Value
Gender (M: F)	159: 79	39: 22	0.672
Age (yr)	53.430 ± 0.869	58.360 ± 1.682	0.012^*^
Duration (yr)	7.17 ± 0.412	11.28 ± 0.930	0.000^**^
BMI (kg/m^2^)	25.62 ± 0.281	25.33 ± 0.448	0.602
WHR	0.95 ± 0.012	0.94 ± 0.019	0.650
FPG (mmol/L)	8.95 ± 0.297	9.37 ± 0.607	0.553
Fasting C-peptide (ng/ml)	1.90 ± 0.078	1.73 ± 0.131	0.421
C-peptide 2h postprandial (ng/ml)	4.70 ± 0.223	3.62 ± 0.385	0.005^*^
HbA1c (%)	8.74 ± 0.164	9.58 ± 0.309	0.007^*^
TG (mmol/L)	1.91 ± 0.119	2.06 ± 0.257	0.876
TC (mmol/L)	4.74 ± 0.073	4.89 ± 0.152	0.330
LDL-C (mmol/L)	2.84 ± 0.062	2.88 ± 0.117	0.753
HDL-C (mmol/L)	1.06 ± 0.023	1.11 ± 0.049	0.456
UA (umol/L)	326.50 ± 6.43	339.74 ± 11.68	0.341
BUN (mmol/L)	5.40 ± 0.107	7.09 ± 0.372	0.000^**^
CRP (mg/L)	7.19 ± 0.735	5.93 ± 0.329	0.880
Genomic DNA methylation (%)	10.441 ± 0.641	10.309 ± 0.698	0.591
TCSS	6.53 ± 0.303	8.58 ± 0.621	0.003^**^
Alb(g/L)	42.50 ± 0.278	39.52 ± 0.624	0.000^**^
IMT (L) (mm)	0.750 ± 0.175	0.804 ± 0.240	0.214
IMT (R) (mm)	0.708 ± 0.012	0.772 ± 0.016	0.005^**^
SBP (mmHg)	137.317 ± 1.388	144.918 ± 2.119	0.000^**^
DBP (mmHg)	80.743 ± 0.711	81.608 ± 1.099	0.274
DR (Y: N)	19:219	40:20	0.000^**^
DPN (Y: N)	76:89	33: 15	0.006^**^
CP (Y: N)	119: 119	37: 24	0.137
Smoke (Y: N)	69: 169	19: 42	0.742
HBP (Y: N)	112: 126	43: 18	0.001^**^

Data are means ± SEM or numbers of patients. The level of DNA methylation, age, duration of diabetes, WHR, fasting C-peptide, C-peptide 2h postprandial, HbA1c, FPG, CRP, TG, Cr, BUN, Alb, 24h-UTP, UACR and IMT were non-parametric and Mann-Whitney U test was used to compare them between two groups. **p < 0.01, *p < 0.05. p values for differences between the both groups were obtained by two-sample t-test, Mann-Whitney U test or χ^2^ test.

**Table 6:** DN as dependent variable in multiple logistic regression analysis.

	B	Std.Error	*p* Value	OR	95% Confidence Interval for OR	
					Lower Bound	Upper Bound
Constant	-0.536	2.170	0.805	0.585	–	–
HbA1c (%)	0.160	0.079	0.042*	1.173	1.006	1.369
BUN (mmol/L)	0.222	0.098	0.023*	1.249	1.030	1.513
DR	0.922	0.352	0.009**	2.514	1.260	5.016
HBP	1.032	2.170	0.009**	2.807	1.297	6.077

In multiple logistic regression analysis, DN, as dependent variable, and the other 12 variables, age, duration, HbA1c, BUN, TCSS, Alb, IMT(R), SBP, 2h postprandial C-peptide, the presence of DR, DPN and HBP, as independent variables, were included in the same model. Only 4 variables, HbA1c, BUN, DR, and HBP were the risk factors of DN (**p < 0.01, *p < 0.05).

#### 3.2.4 Diabetic Peripheral Neuropathy

**Figure 1:**
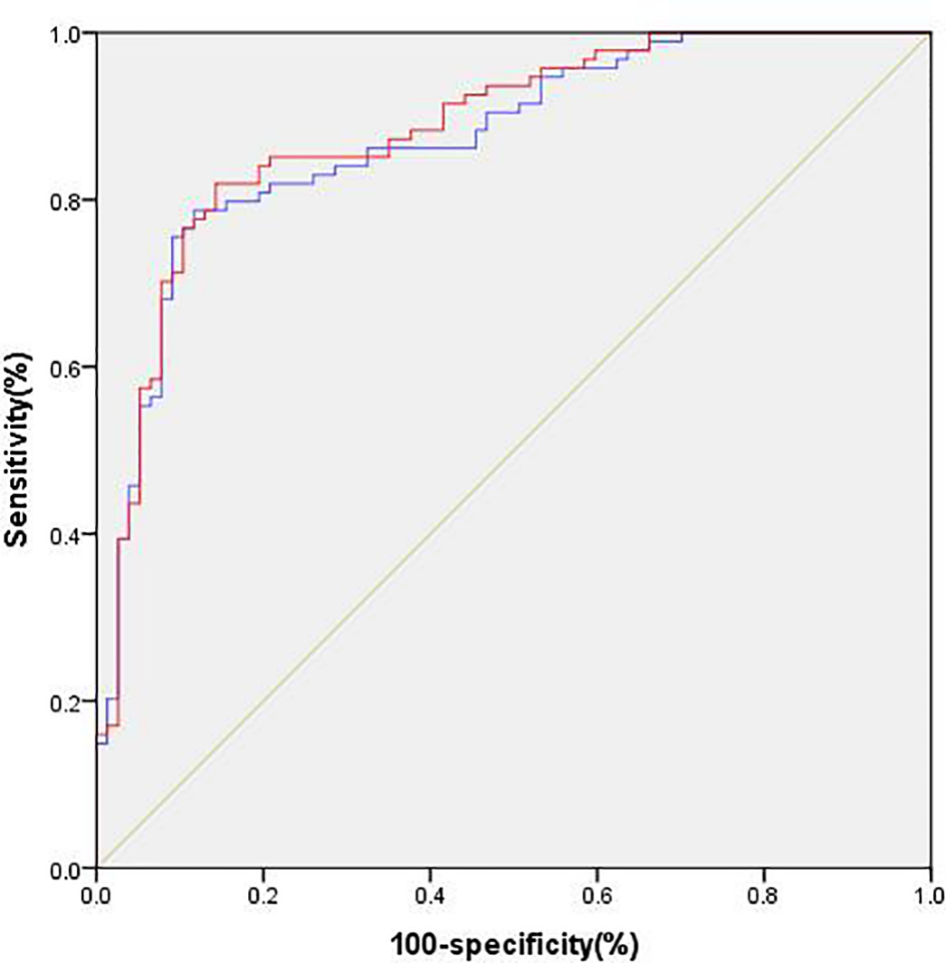
Predictive ability of different models of DPN. Model 1 (blue), conventional model including age, duration, TG, TC, LDL-C, Cr, UA, BUN, eGFR, SBP, the presence of DR and DN. Model 2 (red), model 1 plus the level of genomic DNA methylation.

As shown in **Table 7**, the patients were divided into the DPN group and the non-DPN group. The characteristics as follows were significantly higher in the DPN group: age, duration, TG, TC, LDL-C, Cr, UA, BUN and SBP (**p < 0.01, *p < 0.05, compared with non-DPN, two-sample t-test and Mann-Whitney *U* test). The levels of genomic DNA methylation and eGFR were significantly lower in the DPN group (**p<0.01, *p<0.05, compared with non-DPN, Mann-Whitney *U* test). The presence of DPN was associated with the presence of DR and DN (**p < 0.01, *p < 0.05, *χ*2 test). In order to explore the predictive ability of genomic DNA methylation in DPN, we established two multiple logistic regression models and performed ROC analysis. In the first multiple logistic regression analysis, we set the presence or absence of DPN as a dependent variable, and the other twelve variables except for the level of genomic DNA methylation as independent variables. The findings revealed that age, duration, LDL-C, UA, and the presence of DR were still independently associated with the risk of DPN, while TC, Cr, BUN, eGFR, CRP, SBP, and the presence of DN were no longer related (**Table 8**, **p < 0.01, *p < 0.05, *p*>0.05). The area under the ROC curve (AUC) for the five conventional risk factors was 0.872, as shown in **Figure 1**. In the second multiple logistic regression analysis, we set the presence or absence of DPN as a dependent variable, and the other thirteen variables including the level of genomic DNA methylation as independent variables. The results showed that the level of genomic DNA methylation was also still independently associated with the risk of DPN (**Table 9**, **p < 0.01, *p < 0.05). Introducing the genomic DNA methylation level into the model statistically increased the AUC to 0.883. The AUC for composite factors was greater than any single conventional risk factors, with a sensitivity of 81.9%, specificity of 85.7%, as shown in **Figure 1**.

**Table 7:** Clinical and biochemical characteristics in DPN group and non-DPN group.

Variables	Non-DPN (n=86)	DPN (n=100)	*p* Value
Gender (M: F)	52: 34	67: 33	0.355
Age (yr)	56.870 ± 1.310	63.030 ± 1.207	0.001**
Duration (yr)	5.819 ± 0.551	12.472 ± 0.752	0.000**
BMI (kg/m^2^)	25.407 ± 0.401	25.060 ± 0.283	0.471
WHR	0.944 ± 0.005	0.940 ± 0.006	0.423
FPG (mmol/L)	8.536 ± 0.517	9.319 ± 0.491	0.275
HbA1c (%)	8.612 ± 0.289	8.819 ± 0.234	0.575
Fasting C-peptide (ng/ml)	1.880 ± 0.115	1.890 ± 0.118	0.950
C-peptide 2 hours postprandial (ng/ml)	4.858 ± 3.40	4.493 ± 0.330	0.508
TG (mmol/L)	1.527 ± 0.163	1.968 ± 0.140	0.007**
TC (mmol/L)	4.426 ± 0.135	4.865 ± 0.114	0.013*
LDL-C (mmol/L)	2.549 ± 0.096	2.954 ± 0.098	0.004**
HDL-C (mmol/L)	1.102 ± 0.031	1.074 ± 0.032	0.363
Cr (umol/L)	63.800 ± 2.204	78.340 ± 3.352	0.001**
UA (umol/L)	303.150 ± 10.737	346.350 ± 10.425	0.001**
BUN (mmol/L)	5.555 ± 0.210	6.409 ± 0.240	0.009**
eGFR (ml/min/1.73m^2^)	113.719 ± 3.278	95.108 ± 3.414	0.000**
UACR (mg/g)	218.321 ± 119.476	340.638 ± 123.155	0.159
CRP (mg/L)	5.452 ± 0.070	6.998 ± 1.403	0.311
Genomic DNA methylation (%)	11.361 ± 0.783	9.244 ± 0.556	0.015*
Alb (g/L)	42.002 ± 0.521	41.041 ± 0.449	0.119
24h-UTP (g/24h urine)	171.5 ± 75.758	306.596 ± 88.895	0.234
IMT (L) (mm)	0.80 ± 0.002	0.84 ± 0.003	0.538
IMT (R) (mm)	0.760 ± 0.002	0.78 ± 0.002	0.606
SBP (mmHg)	136.51 ± 1.970	147.240 ± 2.350	0.002**
DBP (mmHg)	78.78 ± 1.16	79.58 ± 1.06	0.612
DR (Y: N)	11: 92	36: 73	0.000**
DKD (Y: N)	15: 89	33: 76	0.006**
CP (Y: N)	15: 89	70: 39	0.908
Smoke (Y: N)	31: 73	36: 73	0.613
HBP (Y: N)	53: 51	71: 38	0.082

Data are means ± SEM or numbers of patients. The level of DNA methylation, duration of diabetes, WHR, fasting C-peptide, C-peptide 2h postprandial, HbA1c, FPG, CRP, TG, Cr, BUN, Alb, eGFR, 24h-UTP, UACR and IMT were non-parametric and Mann-Whitney U test was used to compare them between two groups. **p < 0.01, *p < 0.05. p values for differences between the both groups were obtained by two-sample t-test, Mann-Whitney U test or χ^2^ test.

**Table 8:** DPN as dependent variable in multiple logistic regression analysis.

	B	Std.Error	*p* Value	OR	95% Confidence Interval for OR	
					Lower Bound	Upper Bound
Constant	-9.618	1.879	0.000*	0000	–	–
Age (yr)	0.044	0.018	0.013*	1.045	1.010	1.083
Duration (yr)	0.180	0.041	0.000*	1.197	1.104	1.297
UA (umol/L)	0.006	0.002	0.005**	1.006	1.002	1.010
LDL-C (mmol/L)	1.255	0.292	0.000**	3.508	1.981	6.211
DR	1.570	0.578	0.007**	4.807	1.549	14.913

In multiple logistic regression analysis, DPN, as dependent variable, and the other 12 variables, age, duration, TG, TC, LDL-C, Cr, UA, BUN, eGFR, SBP, the presence of DR and DN, as independent variables, were included in the same model. 6 variables, age, duration, TG, LDL-C and the presence of DR were the risk factors of DPN (**p < 0.01, *p < 0.05).

**Table 9:** DPN as dependent variable in multiple logistic regression analysis.

	B	Std.Error	*p* Value	OR	95% Confidence Interval for OR	
					Lower Bound	Upper Bound
Constant	-8.670	1.884	0.000*	0000	–	–
Age (yr)	0.044	0.018	0.013**	1.045	1.009	1.082
Duration (yr)	0.179	0.041	0.000*	1.195	1.103	1.296
UA (umol/L)	0.005	0.002	0.019**	1.005	1.001	1.009
LDL-C (mmol/L)	1.270	0.291	0.000**	3.561	2.014	6.296
Genomic DNA methylation (%)	-6.917	3.655	0.041*	0.001	0.000	1.279
DR	1.683	0.609	0.006**	5.380	1.631	17.748

In multiple logistic regression analysis, DPN, as dependent variable, and the other 13 variables, age, duration, TG, TC, LDL-C, Cr, UA, BUN, eGFR, SBP, the presence of DR and DN, the level of genomic DNA methylation, as independent variables, were included in the same model. 6 variables, age, duration, TG, LDL-C, DR and the level of genomic DNA methylation were the risk factors of DPN (**p < 0.01, *p < 0.05).

### 3.3 The Influencing Factors of Genomic DNA Methylation

To investigate the factors that influenced genomic DNA methylation, we performed further correlation analysis among the genomic DNA methylation, clinical characteristics, and TCSS scores, and the results were shown in **Table 10**. The genomic DNA methylation level was negatively correlated with BMI, TG and TCSS, and the correlation coefficients (r values) were -0.189, -0.152, and -0.278, respectively (∗*p*<0.05, ∗∗*p*<0.01). In the multiple linear regression analysis, we set the genomic DNA methylation level as a dependent variable and the other 3 variables as independent variables. TCSS and BMI were the risk factors of genomic DNA methylation. Unstandardized coefficients for BMI was -0.069(-0.111, -0.027) and for TCSS was -0.303 (-0.443, -0.164), respectively (**Table 11**, ∗∗*p*<0.01, *p < 0.05). T2DM patients with higher BMI and TCSS scores had lower levels of genomic DNA methylation.

**Table 10:** Correlation analysis among genomic DNA methylation and the other variables.

Variables		BMI	TG	TCSS
Genomic of DNA methylation (%)	r	-0.189	-0.152	-0.278
	*p*	0.01*	0.039*	0.000**

The r value indicated the 3 variables, BMI, TG, TCSS, were likely to be related to the level of genomic of DNA methylation (**p < 0.01, *p < 0.05).

**Table 11:** Genomic DNA methylation as dependent variable in multiple linear regression analysis.

	Unstandardized coefficients		Standardized coefficients	T	*p* Value	95% Confidence Interval for B	
	B	Std.Error	Beta			Lower Bound	Upper Bound
BMI (kg/m^2^)	-0.069	0.021	-0.226	-3.241	0.001**	-0.111	-0.027
TCSS	-0.303	0.071	-0.299	-4.294	0.000**	-0.443	-0.164

In multiple linear regression analysis, genomic of DNA methylation, as dependent variable, and the other 3 variables, BMI, TG, TCSS, as independent variables, were included in the same model. Only two variables, BMI and TCSS were the risk factors of the level of genomic DNA methylation (**p < 0.01).

## 4 Discussion

Diabetes mellitus is estimated to affect 380 million people worldwide by 2025 (7). The disease leads to macrovascular and microvascular complications, including diabetic macroangiopathy, diabetic nephropathy, diabetic retinopathy, and diabetic peripheral neuropathy. Chronic complications of diabetes negatively affect the quality of patients’ life. It is important to study the clinical characteristics and the risk factors of diabetic complications, providing potential targets for clinical treatments.

Cardiovascular disease is considered to be the most common cause of disability and death in patients with diabetes. Pathophysiology is thrombus, plaque ulceration, luminal stenosis or bleeding from atherosclerosis. Cervical atherosclerosis is considered to be the most important risk factor for acute ischemic cerebrovascular disease. Therefore, in this study, we analyzed the related risk factors of carotid atherosclerosis in patients with diabetes. As shown in the results, gender, age and BMI were independently associated with the risk of CP, consistent with the most studies (8–10). However, there was no difference in lipid parameters, such as TC, TG, and LDL-C, between the CP group and the non-CP group, which might be on account of the interference of lipid-lowering drugs taken by some patients when they were admitted to the hospital.

Diabetic retinopathy, diabetic nephropathy and diabetic peripheral neuropathy are all microvascular complications, which have common pathogenesis including hyperglycemia, polyol metabolic abnormalities, protein non-enzyme glycation, cytokines, free radical action, inflammatory media oxidative stress and genetic background (11). So, patients with one microvascular complication were more likely to have other microvascular complications (12). The results of this study are consistent with the above conclusion. Growing clinical studies have explored the risk factors for microvascular complications in diabetes. Most studies discovered that the prevalence of diabetic microvascular complications increased with age and duration (13). Hypertension and lipid metabolism disorders has a promoting effect on the development of microvascular lesion in diabetes (14, 15). Another widely recognized factor is HbA1c, which can be used as an important indicator for blood glucose levels and diabetic microvascular complications (16). Serum albumin was also reported to be closely related to microvascular complications of diabetes and considered as a marker of chronic inflammation and oxidative stress in DM patients (16). Similar results were all obtained in this study.

Interestedly, we found that the level of genomic DNA methylation might be an independent risk factor of DPN. DPN is considered to be affected by both genetic and lifestyle factors. At present, epigenetic mechanisms, such as DNA methylation, RNA regulation, histone modifications, and chromatin remodeling, have emerged as a potential link between environmental factors and gene expression (17). DNA methylation affects gene expression and is an essential epigenetic process. Hyperglycemia causes changes in DNA methylation, which in turn alter the expression of genes and many key molecules, ultimately leading to chronic complications of diabetes (18). Assessment of DNA methylation status includes gene-specific and genome-wide DNA methylation analysis. We previously found that P2x3r gene in dorsal root ganglion neurons was significantly demethylated, upregulating the expression of the P2X3 receptor and leading to diabetic painful neuropathy in rats with diabetes (19). We also found that DNA demethylation of cystathionine-β-synthetase (CBS), an endogenous H_2_S synthase, was involved in diabetic gastric hypersensitivity (20). A comprehensive analysis of DNA methylation profiles of sural nerves in DPN patients also suggests the important role of DNA methylation in DPN progression (21). We wondered whether the level of genomic DNA methylation in leukocytes was associated with DPN. In this study, we found that the genomic DNA methylation level was significantly lower in DPN patients. The logistic regression analysis showed that the level of genomic DNA methylation was an independent risk factor of DPN.

Studies have shown links between DNA methylation and other chronic complications of diabetes. Abnormal DNA methylation in the proximal tubules was reported to be associated with DN (22). Elisabet et al. found that genome-wide DNA methylation level in blood could be considered as a predictive biomarker for DR (23). In the present study, we meant to figure out whether genomic DNA methylation is a influencing factor for DPN or for all chronic complications of diabetes, including DR, DPN, DN and CP. We grouped the population according to different complications and found that the genomic DNA methylation level was significantly higher in patients with CP, but didn’t change in patients with DN and DR. Taking CP as a dependent variable, the logistic regression analysis showed that the level of genomic DNA methylation was not an independent risk factor of CP. Based on the findings, we inferred that genomic DNA methylation is a relatively specific risk factor for DPN. In addition, our results suggested that incorporating the level of genomic DNA methylation into the prediction model could improve the ability to predict DPN on the basis of conventional risk factors.

To further clarify the relationship between DPN and genomic DNA methylation, we analyzed the factors that influenced genomic DNA methylation using the correlation analysis and multiple stepwise regression analysis. In addition to TCSS, BMI was found to be another negative influencing factor for genomic DNA methylation. The result was consistent with previous researches. Miina Ollikainen et al. found significant differences in DNA methylation between twins, when the heavier co-twins had excess liver fat (24). Wahl S. found that DNA methylation status in leukocytes changed in obesity associated with metabolic disorders (25). These studies confirm that obesity is a factor that causes changes in DNA methylation. In the presence study, BMI and WHR were not the influencing factor of DPN, so obesity or not did not interfere with the effect of genomic DNA methylation on DPN.

What is the link between DPN and genomic DNA methylation? It is well known that DNA methylation status is affected by factors such as environment, age, gender, and diseases, and is always in the process of dynamic change. DNA methylation may vary between different cells, tissues or individuals, and even between different periods of development of the same individual. In general, CpG island, located in the gene promoter region, is in a methylated state, which often leads to a reduction in the gene expression. The higher the degree of DNA methylation, the lower its transcription activity, and the methylation in the part of the gene regulates the extension and slicing of gene transcription (26). Evidence from both the Diabetes Control and Complications Trial has revealed that long-term hyperglycemia could lead to persistent diabetic complications even after better control was established, which was well known as the legacy effect or the metabolic memory phenomenon (27). Because of the existence of “metabolic memory”, the treatment of complications of diabetes is very difficult. Ansgar S. Olsen’s data showed that metabolic memory is heritable and that the inheritance is associated with DNA demethylation and abnormal gene expression induced by hyperglycemia. In our present study, the results show that the lower the level of genomic DNA methylation, the higher TCSS scores, suggesting that patients with lower genomic DNA methylation level were more likely to develop to DPN. It was reported that folic acid levels in diabetes patients’ blood was associated with reduced genomic DNA methylation (28). Long-term vitamin B12 and folic acid treatment was demonstrated to alter the genomic DNA methylation level in patients (29). Vitamin B12 and folic acid deficiency play important roles in accelerating diabetic neuropathy, while they are biomarkers of one-carbon metabolism. We hypothesize that one-carbon unit deficiency in diabetic patients results in genomic DNA demethylation, which in turn stimulates the activation of key genes and activates additional signal pathways that lead to DPN.

This study had some limitations. Firstly, the study was cross-sectional and therefore could not longitudinally observe the relationship between genomic DNA methylation and the evolution of diabetic complications. Secondly, the sample size of the study was small, and the selected patients were hospitalized ones, which might cause bias. Therefore, multicenter studies with larger sample are required. Thirdly, we speculated that vitamin B12 and folic acid affected the genomic DNA methylation level, but we didn’t test the levels of vitamin B12 and folic acid.

## 5 Conclusion

As the above results have shown, low level of genomic DNA methylation is a relatively specific risk factor for diabetic peripheral neuropathy in patients with type 2 diabetes, rather than a specific risk factor for other chronic complications of diabetes, such as DN, DR or CP.

## Data Availability Statement

The original contributions presented in the study are included in the article/supplementary material. Further inquiries can be directed to the corresponding authors.

## Ethics Statement

The studies involving human participants were reviewed and approved by the Institutional Review Board of the Second Affiliated Hospital of Soochow University. The patients/participants provided their written informed consent to participate in this study.

## Author Contributions

XW, WY, YZ collected and analyzed the data and wrote the article. SZ, MJ collected and analyzed the data. JH reviewed and edited the article. H-HZ designed and supervised the study and edited the article. H-HZ or JH is the guarantor of this study and, as such, had full access to all the data in the study and takes responsibility for the authenticity of the data and the accuracy of the data analysis. All authors contributed to the article and approved the submitted version.

## Funding

This work was supported by grants from the National Natural Science Foundation of China (82071234 to H-HZ, 82170836 to JH) and from the Jiangsu Youth Medical Talents Project (QNRC2016874 to H-HZ).

## Conflict of Interest

The authors declare that the research was conducted in the absence of any commercial or financial relationships that could be construed as a potential conflict of interest.

## Publisher’s Note

All claims expressed in this article are solely those of the authors and do not necessarily represent those of their affiliated organizations, or those of the publisher, the editors and the reviewers. Any product that may be evaluated in this article, or claim that may be made by its manufacturer, is not guaranteed or endorsed by the publisher.

## Abbreviations

T2DM, type 2 diabetes mellitus; CP, Carotid plaque; AS, Atherosclerosis; DN, Diabetic Nephropathy; DR, Diabetic Retinopathy; DPN, Diabetic peripheral neuropathy; TCSS, Toronto Clinical Scoring System; HHS, hyperglycemic hyperosmolar syndrome; DKA, diabetic ketoacidosis; DBP, Diastolic blood pressure; SBP, Systolic blood pressure; BMI, Body mass index; WHR, Waist-to-hip ratio; FPG, Fasting plasma glucose; HbA1c, Glycated hemoglobin; TG, Triglyceride; TC, Total cholesterol; LDL-C, Low density lipoprotein; HDL-C, High density lipoprotein; Cr, Creatinine; BUN, Blood urea nitrogen; UA, Uric acid; Alb, Albumin; CRP, C-reactive protein; 24-hUTP, 24-hour urine total protein; UACR, Urine microalbumin creatinine ratio; eGFR, Estimated glomerular filtration rate; IMT, Intima-media thickness; LC-MS/MS, Liquid chromatography-tandem mass spectrometry.

## References

[1] Sinclair A Saeedi P Kaundal A Karuranga S Malanda B Williams R. Diabetes and Global Ageing Among 65-99-Year-Old Adults: Findings From the International Diabetes Federation Diabetes Atlas, 9 Edition. Diabetes Res Clin Pract (2020) 162:108078. doi: 10.1016/j.diabres.2020.108078 32068097

[2] Raciti G Nigro C Longo M Parrillo L Miele C Formisano P. Personalized Medicine and Type 2 Diabetes: Lesson From Epigenetics. Epigenomics (2014) 6:229–38. doi: 10.2217/epi.14.10 24811791

[3] Binns-Hall O Selvarajah D Sanger D Walker J Scott A Tesfaye S. One-Stop Microvascular Screening Service: An Effective Model for the Early Detection of Diabetic Peripheral Neuropathy and the High-Risk Foot. Diabetic Med J Br Diabetic Assoc (2018) 35:887–94. doi: 10.1111/dme.13630 PMC6033008 29608799

[4] Zhang H Han X Wang M Hu Q Li S Wang M. The Association Between Genomic DNA Methylation and Diabetic Peripheral Neuropathy in Patients With Type 2 Diabetes Mellitus. J Diabetes Res (2019) 2019):2494057. doi: 10.1155/2019/2494057 31781662 PMC6875377

[5] Bril V Perkins B. Validation of the Toronto Clinical Scoring System for Diabetic Polyneuropathy. Diabetes Care (2002) 25:2048–52. doi: 10.2337/diacare.25.11.2048 12401755

[6] Touboul P Hennerici M Meairs S Adams H Amarenco P Bornstein N. Mannheim Carotid Intima-Media Thickness and Plaque Consensus (2004-2006-2011). An Update on Behalf of the Advisory Board of the 3rd, 4th and 5th Watching the Risk Symposia, at the 13th, 15th and 20th European Stroke Conferences, Mannheim, Germany, 2004, Brussels, Belgium, 2006, and Hamburg, Germany, 2011. Cerebrovascular Dis (Basel Switzerland) (2012) 34:290–6. doi: 10.1159/000343145 PMC3760791 23128470

[7] Tarr J Kaul K Chopra M Kohner EM Chibber R. Pathophysiology of Diabetic Retinopathy. ISRN Ophthalmol (2013) 2013:343560. doi: 10.1155/2013/343560 24563789 PMC3914226

[8] van der Heijden D van Leeuwen M Janssens G Lenzen MJ van de Ven PM Eringa EC. Body Mass Index is Associated With Microvascular Endothelial Dysfunction in Patients With Treated Metabolic Risk Factors and Suspected Coronary Artery Disease. J Am Heart Assoc (2017) 6(9):e006082. doi: 10.1161/JAHA.117.006082 28912211 PMC5634274

[9] Rovella V Anemona L Cardellini M Saggini A Santeusanio G Bonanno E. The Role of Obesity in Carotid Plaque Instability: Interaction With Age, Gender, and Cardiovascular Risk Factors. Cardiovasc Diabetol (2018) 17:46. doi: 10.1186/s12933-018-0685-0 29598820 PMC5874994

[10] Vlachopoulos C Aznaouridis K Stefanadis C. Prediction of Cardiovascular Events and All-Cause Mortality With Arterial Stiffness: A Systematic Review and Meta-Analysis. J Am Coll Cardiol (2010) 55:1318–27. doi: 10.1016/j.jacc.2009.10.061 20338492

[11] Ahsan H. Diabetic Retinopathy–Biomolecules and Multiple Pathophysiology. Diabetes Metab syndrome (2015) 9:51–4. doi: 10.1016/j.dsx.2014.09.011 25450817

[12] Al-Rubeaan K Abu El-Asrar AM Youssef AM Subhani SN Ahmad NA Al-Sharqawi AH. Diabetic Retinopathy and Its Risk Factors in a Society With a Type 2 Diabetes Epidemic: A Saudi National Diabetes Registry-Based Study. Acta Ophthalmologica (2015) 93:E140–7. doi: 10.1111/aos.12532 25270515

[13] Pai Y Lin C Lee I Chang MH. Prevalence and Biochemical Risk Factors of Diabetic Peripheral Neuropathy With or Without Neuropathic Pain in Taiwanese Adults With Type 2 Diabetes Mellitus. Diabetes Metab syndrome (2018) 12:111–6. doi: 10.1016/j.dsx.2017.09.013 29042249

[14] Iqbal Z Bashir B Ferdousi M Kalteniece A Alam U Malik RA. Lipids and Peripheral Neuropathy. Curr Opin Lipidol (2021) 32:249–57. doi: 10.1097/MOL.0000000000000770 34101657

[15] Kazamel M Stino A Smith A. Metabolic Syndrome and Peripheral Neuropathy. Muscle Nerve (2021) 63:285–93. doi: 10.1002/mus.27086 33098165

[16] Yaribeygi H Atkin S Sahebkar A. A Review of the Molecular Mechanisms of Hyperglycemia-Induced Free Radical Generation Leading to Oxidative Stress. J Cell Physiol (2019) 234:1300–12. doi: 10.1002/jcp.27164 30146696

[17] Maunakea A Chepelev I Zhao K. Epigenome Mapping in Normal and Disease States. Circ Res (2010) 107:327–39. doi: 10.1161/CIRCRESAHA.110.222463 PMC2917837 20689072

[18] Hao J Hua L Fu X Zhang X Zou Q Li Y. Genome-Wide DNA Methylation Analysis of Human Peripheral Blood Reveals Susceptibility Loci of Diabetes-Related Hearing Loss. J Hum Genet (2018) 63:1241–50. doi: 10.1038/s10038-018-0507-y 30209346

[19] Zhang H Hu J Zhou Y Qin X Song ZY Yang PP. Promoted Interaction of Nuclear Factor-κb With Demethylated Purinergic P2X3 Receptor Gene Contributes to Neuropathic Pain in Rats With Diabetes. Diabetes (2015) 64:4272–84. doi: 10.2337/db15-0138 26130762

[20] Zhang H Hu J Zhou Y Hu S Wang YM Chen W. Promoted Interaction of Nuclear Factor-κb With Demethylated Cystathionine-β-Synthetase Gene Contributes to Gastric Hypersensitivity in Diabetic Rats. J Neurosci Off J Soc Neurosci (2013) 33:9028–38. doi: 10.1523/JNEUROSCI.1068-13.2013 PMC6705038 23699514

[21] Guo K Elzinga S Eid S Figueroa-Romero C Hinder LM Pacut C. Genome-Wide DNA Methylation Profiling of Human Diabetic Peripheral Neuropathy in Subjects With Type 2 Diabetes Mellitus. Epigenetics (2019) 14:766–79. doi: 10.1080/15592294.2019.1615352 PMC6615525 31132961

[22] Marumo T Yagi S Kawarazaki W Nishimoto M Ayuzawa N Watanabe A. Diabetes Induces Aberrant DNA Methylation in the Proximal Tubules of the Kidney. J Am Soc Nephrol JASN (2015) 26:2388–97. doi: 10.1681/ASN.2014070665 PMC4587689 25653098

[23] Agardh E Lundstig A Perfilyev A Volkov P Freiburghaus T Lindholm E. Genome-Wide Analysis of DNA Methylation in Subjects With Type 1 Diabetes Identifies Epigenetic Modifications Associated With Proliferative Diabetic Retinopathy. BMC Med (2015) 13:182. doi: 10.1186/s12916-015-0421-5 26248552 PMC4527111

[24] Ollikainen M Ismail K Gervin K Kyllönen A Hakkarainen A Lundbom J. Genome-Wide Blood DNA Methylation Alterations at Regulatory Elements and Heterochromatic Regions in Monozygotic Twins Discordant for Obesity and Liver Fat. Clin Epigenet (2015) 7:39. doi: 10.1186/s13148-015-0073-5 PMC4393626 25866590

[25] Wahl S Drong A Lehne B Loh M Scott WR Kunze S. Epigenome-Wide Association Study of Body Mass Index, and the Adverse Outcomes of Adiposity. Nature (2017) 541:81–6. doi: 10.1038/nature20784 PMC5570525 28002404

[26] Jones P. Functions of DNA Methylation: Islands, Start Sites, Gene Bodies and Beyond. Nat Rev Genet (2012) 13:484–92. doi: 10.1038/nrg3230 22641018

[27] Intine R Sarras M. Metabolic Memory and Chronic Diabetes Complications: Potential Role for Epigenetic Mechanisms. Curr Diabetes Rep (2012) 12:551–9. doi: 10.1007/s11892-012-0302-7 PMC3432745 22760445

[28] Nilsson E Matte A Perfilyev A de Mello VD Käkelä P Pihlajamäki J. Epigenetic Alterations in Human Liver From Subjects With Type 2 Diabetes in Parallel With Reduced Folate Levels. J Clin Endocrinol Metab (2015) 100:E1491–501. doi: 10.1210/jc.2015-3204 PMC4702449 26418287

[29] Yadav D Shrestha S Lillycrop K Joglekar CV Pan H Holbrook JD. Vitamin B Supplementation Influences Methylation of Genes Associated With Type 2 Diabetes and Its Intermediate Traits. Epigenomics (2018) 10:71–90. doi: 10.2217/epi-2017-0102 29135286

